# Perceptions related to the layout of Visual Abstracts among physicians and medical students

**DOI:** 10.1590/2175-8239-JBN-2024-0146en

**Published:** 2024-12-16

**Authors:** Pedro Cesana Portugal, José A. Moura-Neto

**Affiliations:** 1 Escola Bahiana de Medicina e Saúde Pública, Salvador, BA, Brazil.

**Keywords:** Abstracts, Visual Perception, Aesthetics, Color, Medical Illustration

## Abstract

**Introduction:**

Visual Abstract is a visual summary of the most relevant information from a scientific article, presented as an infographic. Despite the growing use of Visual Abstracts by journals around the world, studies evaluating their components to guide their development remain scarce.

**Objective:**

The primary objective of this study is to identify the aesthetic perceptions of Visual Abstracts components by physicians and medical students.

**Methods:**

Cross-sectional study, using a virtual questionnaire sent via email to a convenience sample comprising physicians and medical students. Data were analyzed using descriptive statistics, with means and standard deviation or median and interquartile range, depending on the type of the variable distribution. Categorical variables are presented in absolute and relative numbers.

**Result:**

The research sample consisted mainly of medical students (65%), who were female (57.2%), with a median age of 23.5 years (IQR 21–42.25). The majority of respondents declared no prior knowledge on Visual Abstracts (61.7%). Of the analyzed variables, preferences included icons (56.7%), in a monochrome style (36.7%), second-dimensional (81.1%), and moderately detailed layout (56.7%), using the “original” color (91.7%), and structured in IMRaD format (73.9%).

**Conclusion:**

Several visual components influence the aesthetic perception of physicians and medical students regarding Visual Abstracts, with particular emphasis on textual objectivity, clarity of colors, and the use of icons.

## Introduction

The Visual Abstract (VA), also known as Graphical Abstract, is a visual synthesis of the most relevant information, from a scientific article, presented as an infographic. Its purpose is to convey a message that could be easily handled and understandable. VA is not intended to replace reading the article, but rather to assist the reader in deciding whether or not to proceed with a full reading^1,2,3,4^.

Despite its increasing use by international journals, studies remain rare and are limited to understanding VAs in the dissemination of knowledge. Annals of Surgery has published VAs since 2016. In Brazil, the Brazilian Journal of Nephrology was a pioneer in adopting the tool, having started in 2018. However, there is a lack of information regarding layout components, visual design, and the aesthetic perception of users that could guide editors and authors in developing VAs.

The VA can be designed from various elements, such as the summary of results, title, layout, publication journal, and the “making it visual”, which corresponds to the use of icons, pictograms, and images in the design, rendering it more illustrative. This visual aspect, often regarded as the most complex and challenging part of VA construction, due to the complexity of selecting appropriate colors and formats, still lacks a defined aesthetic pattern to be used among VA editors and authors. Thus, journals plan the “making it visual” without first ensuring a favorable visual aesthetic, which can result in conflicting aesthetic perceptions to the VA’s style, potentially rendering it less attractive and less effective in engaging readers with the full article^3,4,5,6,7,8,9^.

The primary objective of this study is to identify the aesthetic perceptions of the components of VA layouts among physicians and medical students when exposed to different visual designs. The secondary objectives are to identify the prior knowledge of the sample regarding VA, and to recognize the visual components of the layout that most influence the choice of the aesthetic pattern for a VA.

## Methods

### Design and Sampling

This was a cross-sectional, convenience sampling study conducted from February to July 2022 at the *Escola Bahiana de Medicina e Saúde Pública* (EBMSP). Medical students and physicians aged 18 years or older were included, while those who did not complete the Informed Consent Form (ICF) were excluded.

### Data Collection

Data were collected using a single fill-in form on Google Forms, made available for completion through the academic email of medical students from the 1st to the 12th semester, and physicians at EBMSP. Data collection was conducted after approval by the Research Ethics Committee. A detailed evaluation of the data stored in a Microsoft Excel spreadsheet was then performed. The databases were reviewed and fed in solely by the researchers responsible for the study.

**Figure 1.**
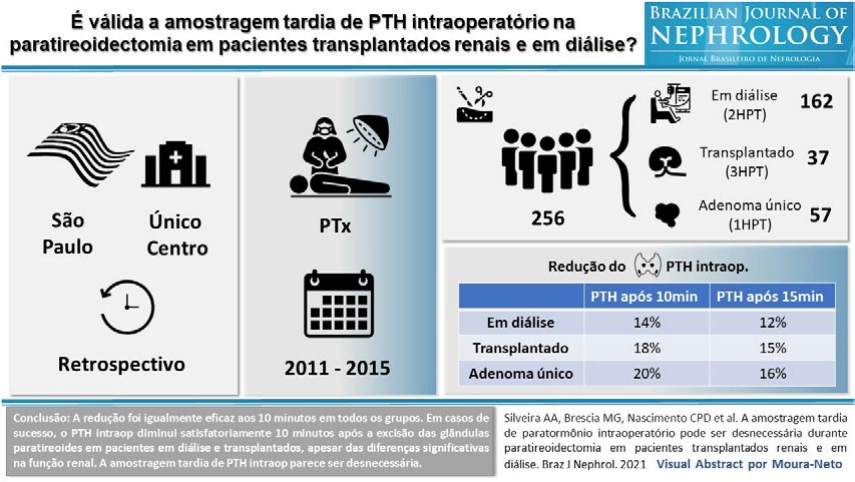
Visual Abstract used as a base model, published in the Brazilian Journal of Nephrology in 2021; 43(2):228–35.

The form was created by the researchers themselves. The VA that served as the base model was developed by the study researcher himself, drawing on a VA published by the Brazilian Journal of Nephrology (BJN) in 2021 (43(2):228–35), with the approval of the journal’s editors (Figure 1). Based on this model, the color used was designated as “Original”; the icon as “Monochrome”; dimensionality in 2D; and the font type Arial. The layout was defined as “Unstructured” due to the absence of a clearly described Introduction – Methodology – Results – Discussion/Conclusion (IMRD) structure; the border of the box containing the information was called “Rectangular”, due to the presence of vertices; and the number of divisions of the VA content, “Quadruple”, due to the presence of four subdivisions. The use of medical term abbreviations was labeled as “With Abbreviations”; and the footer layout used as “Based on BJN and Clinical Journal of the American Society of Nephrology (CJASN)”, since these journals tend to follow this pattern in the VA construction; the details division was on the basis of the Likert Scale (a scale of an ordinal nature with verbal descriptions containing different levels of opinions).

The VA design was modified and (re)created in PowerPoint. From it, the Hexadecimal color codes were obtained, and the Adobe Color application was used to modify the color of the visual summary. The alteration was based on the color harmony suggested by the application, choosing the “Analogous”, “Monochrome”, or “Shadow” patterns. ‘Analogous’ corresponds to the use of adjacent colors on the color wheel; ‘Monochrome’ refers to the use of a single color with variations in saturation and brightness; and ‘Shadow’ involves using five colors with the same hue and saturation but different brightness^10,11^.

The icons were selected from the “Flaticons”, “Noun Project” and “Icon Finder” websites, and the 3D transformation was done using the “Tinkercad” website. The icon style varied among “Monochrome”, “Flat Color”, and “Outlined Color”. The “Mono­chrome” or “Glyph Icon” is entirely black, with a single coloration. The “Flat Color”, or “Flat Icon”, is a style that employs a combination of different colors. The “Outlined Color” style, or “Lineal Color” consists of strokes and is filled with colors^12,13^.

The images were selected from the “Freepik” and “Istock” websites, varying between pixel and vector images. A pixel image is composed of pixels, defined as the standard unit of measurement for digital images, visible when the image is enlarged. A vector image is reproduced by pixels, but not constructed by them, but rather by lines, curves and filled shapes^14^.

The footer options were chosen based on both international and national journals that were pioneers in the use of VA, such as the BJN, which chooses to place the article’s conclusion in a more descriptive manner, the Annals of Surgery, which places the article’s reference in the footer, and the New England Journal of Medicine (NEJM), which succinctly summarizes the article’s conclusion in the footer of the VA.

### Statistical Analysis

All variables were analyzed for their type of distribution, whether Gaussian or not. This analysis involved obser­ving the degree of similarity between the frequency dis­tribution curves and the Gaussian curve, as well as using the Shapiro-Wilk and/or Kolmogorov-Smirnov tests.

Data analysis was performed using descriptive statistics, with means and standard deviation or median and interquartile range, depending on the type of variable distribution, while categorical variables were presented in absolute and relative numbers.

### Ethical Aspects

This study was approved by the EBMSP Research Ethics Committee on February 11, 2022, under opinion No. 5.238.369. The ICF was used for data collection.

## Results

A total of 181 responses to the questionnaire were obtained between February and July 2022. Of these, 180 (99.4%) responses remained for analysis, after applying the exclusion criteria. The majority of the sample (57.2%) was female, and the median age of physicians and medical students was 23.5 years (IQR 21–42.25). The occupation variable, assessed through the physician and medical student dichotomy, was mostly (65%) represented by students (Table 1).

**Table 1.** Sociodemographic characteristics

Variable	Sample (N = 180)
**Sex**	
Male	77 (42.8%)
Female	103 (57.2%)
**Age (in years)**	23.5 (21–42.25)
**Occupation**	
Physician	63 (35%)
Medical Student	117 (65%)

Source: Qualitative variables were expressed as absolute (valid per­centage), and quantitative variables as median (interquartile range – IQR).

The majority (61.7%) of respondents were unaware of VAs. Among the groups, most physicians were familiar with the tool (59.4%), while around a quarter of students (26.5%) had prior knowledge of VAs (Table 2). Aesthetic perceptions were identified through several variables, with the preferred ones including the title presented as the article’s topic (39.4%), the layout in the original pattern (91.7%), with a preference for the use of icons over images (56.7%), and for the icon in monochrome (36.7%) and two-dimensional (81.1%) styles.

**Table 2.** Previous knowledge of a Visual Abstract

Variable	Sample (N = 180)
**Medical Student**	117 (65%)
Knows VA	31 (26.5%)
Does not know VA	86 (73.5%)
**Physician**	63 (35%)
Knows VA	38 (60.3%)
Does not know VA	25 (39.7%)

Source: Qualitative variables were expressed as absolute numbers (valid percentages).

Furthermore, the IMRaD-structured layout (73.9%) was the most relevant, with no abbreviations (55.6%), Arial font (46.1%), and moderate detail (56.7%). In addition, three or more divisions (47.2%), a rectangular box border (58.9%), and a footer based on the NEJM (43.9%) featured most prominently (Table 3). The component considered most influential in the aesthetic pattern for the VA’s visual design was the detailing of information (83.3%), followed by layout color (81.1%), the use of icons and images (71.7%), title presentation design (70%), and layout structure (69.4%) (Table 4).

**Table 3.** Aesthetic perceptions of the layout components of a Visual Abstract

Variable	Sample (N = 180)
**Title presentation design**	
Research question	59 (32.8%)
Article title	44 (24.4%)
Article topic	71 (39.4%)
No preference	06 (3.3%)
**Layout color**	
Original	165 (91.7%)
Shadow	05 (2.8%)
Analogue	06 (3.3%)
Monochrome	03 (1.7%)
No preference	01 (0.6%)
**Icon style**	
Monochrome	66 (36.7%)
Flat color	56 (31.1%)
Outlined color	51 (28.3%)
No preference	06 (3.3%)
**Others**	
Monochrome or Outlined color	01 (0.6%)
**Icon dimension**	
2D version	146 (81.1%)
3D version	23 (12.8%)
No preference	11 (6.1%)
**Use of icons and other images**	
Icon	102 (56.7%)
Pixel image	15 (8.3%)
Vector image	59 (32.8%)
No preference	04 (2.2%)
**Font type**	
Arial	83 (46.1%)
Consolas	03 (1.7%)
Courier New	06 (3.3%)
Georgia	12 (6.7%)
Times New Roman	25 (13.9%)
Verdana	28 (15.6%)
No preference	23 (12.8%)
**IMRaD structure**	
Non-structured	25 (13.9%)
Structured	133 (73.9%)
No preference	22 (12.2%)
**Closed box border**	
Rectangular	106 (58.9%)
Rounded	58 (32.2%)
No preference	16 (8.9%)
**Number of divisions**	
Single	16 (8.9%)
Double	69 (38.3%)
Three or more	85 (47.2%)
No preference	09 (5%)
**Others**	
Triple division	01 (0.6%)
**Use of abbreviations**	
With abbreviations	68 (37.8%)
No abbreviations	100 (55.6%)
No preference	10 (5.6%)
**Others**	
With abbreviations, as long as there is a legend below explaining it	02 (1.1%)
**Detailing of information**	
Grade 1 = Essential information only	09 (5%)
Grade 2π	19 (10.6%)
Grade 3 = Moderate detailing	102 (56.7%)
Grade 4ππ	34 (18.9%)
Grade 5 = Maximum possible detailing	11 (6.1%)
No preference	05 (2.8%)
**Footer design**	
Based on BJN* and CJASN**	65 (36.1%)
Based on Annals of Surgery***	32 (17.8%)
Based on NEJM	79 (43.9%)
No preference	03 (1.7%)
**Others**	
Based on BJN and CJASN, with summarized conclusion	01 (0.6%)

Source: Questionnaire developed by the authors. Qualitative variables were expressed as absolute numbers (valid percentages). πIntermediate stage between Grades 1 and 3; ππIntermediate stage between Grades 3 and 5; *Brazilian Journal of Nephrology. **Clinical Journal of the American Society of Nephrology. ***New England Journal of Medicine.

**Table 4.** Visual components that most influence the choice of aesthetic design pattern for a Visual Abstract

Variable	Sample (N = 180)
Title presentation design	126 (70%)
Layout color	146 (81.1%)
Icon style	84 (46.7%)
Icon dimension	76 (42.2%)
Use of icons and other images	129 (71.7%)
Font type	113 (62.8%)
IMRaD structure	125 (69.4%)
Closed box border	56 (31.1%)
Number of divisions	101 (56.1%)
Detailing of information	150 (83.3%)
Footer design	87 (48.3%)
None	01 (0.6%)
**Other**	
Contents	01 (0.6%)

Source: Questionnaire developed by the authors. Qualitative variables were expressed as absolute numbers (valid percentages).

## Discussion

The present study demonstrated various components considered influential for the aesthetic pattern of VAs. Despite the lack of knowledge about “making it visual”, the scientific field has primarily focused on comparing the VA with the written abstract for disseminating information. A 2017 study found that the use of VAs increased the number of impressions (times the tweet was viewed), retweets (reposting the tweet to one’s followers), and the average number of inspections to the original article compared to using the article title alone^7^. Another study, published in 2020, showed an increase in impressions, retweets, and clicks with the use of VAs compared to Text Abstracts (screenshot of the abstract available on PubMed)^5^.

VAs have been increasingly used by journals with a high Impact Factor, such as NEJM, Annals of Surgery, CJASN, Journal of the American Society of Nephrology (JASN), and Kidney3604. However, there is no established pattern for the quality of VA visual design, generating a variety of aesthetic constructions without consistent technical support. In this sense, the novelty of this study lies in its approachto the aesthetic perception of VAs, and the recognition of visual components, contributing to guiding editors and authors in the creation of a layout graphic design that is more attractive to readers.

VA layouts combine different elements to ensure their ability to convey and memorize content. Based on the variables in this study, the detailing of information was considered the component with the greatest influence on the aesthetic pattern, with a preference for a moderate level of detailing. Balancing the amount of information in the VA is important to ensure clarity and organization of the layout, and can have an impact on the reader’s perception and the understanding of the study’s results^15,16^. The construction of a layout with a visual narrative associated with the hierarchical arrangement of information in a logical, intuitive and fluid sequence - for instance, from left to right and top to bottom - contributes to the development of visual and textual memory. These are two of the four learning styles in the VARK model - an acronym for Visual, Auditory, Reading/Writing and Kinesthetic learning modalities.

The color of the layout was the second most influential component for the visual design of the VA, with a preference for the “Original” color, corres­ponding to the standard color and color palette of the VA base model used by the journal. Color influences information recognition, readability, and makes the layout visually attractive. Colors could be used in contrast to highlight information, and in associations (such as red, to emphasize a warning) with the aim of drawing the public’s attention to information of greater importance, thereby strengthening long-term memory.

The preference for the “Original” color may result from constant exposure to the base model layout during the questionnaire. It may also be due to the simplicity of combining the colors used, or limited theoretical-practical design skills among the authors, hindering the construction of the VA based on color harmony^17^.

There was a preference for the use of icons over images, and this was considered the third most relevant aesthetic component. The use of icons and images, in general, makes the design more illustrative, capturing the individual’s attention and enhancing recall capacity. In general, icons are smaller and more diverse, with different shapes and styles, which may imply a greater range of choices that suit the construction of an aesthetic design for the VA; however, in most VAs, there are icons in their traditional layout, representing a bias for respondents who are already familiar with the pattern^4,18,19^.

Among the icons, the primary choice was the “Monochrome” style (36.7%). The difference between “Flat Colored” and “Outlined Colored”, however, lies only in the presence of an outline that highlights the perimeter of the pictogram. Nevertheless, when added together, the preference for using colored icons, flat or outlined, accounts for 59.4%, which may suggest a preference for using colors in VAs. Regarding images, the vector version was preferred, possibly due to its ability to achieve shapes that are closer to reality, and to maintain the quality of the image, regardless of size^14^.

The layout structure was considered the fifth most influential component for the aesthetic pattern of a VA. Layouts with symmetry and proportionality generate a sense of order and balance, which reduces cognitive overload and simplifies message retention, making it easier to remember/recall the information. The clear description of the IMRaD, as well as the layout featuring three or more divisions, better distributes and organizes ideas, directing the reader to key points in the text. This has an impact on the effectiveness of conveying information, especially considering that the majority of the sample consists of students who are still in the process of developing their scientific reading and interest^15,20,21^. Despite this, along with the font type, they showed the highest percentage of “No Preference”, which makes the aesthetic relevance of these subtypes debatable.

The preferred title for the VA presentation was in the “Article Topic” format. A visually simpler title can enhance message comprehension, and make the layout more accessible and straightforward, going hand in hand with the influence of information detail on the aesthetics of visual design^15,16^.

Different layouts communicate with different target audiences through the arrangement of components, selection of illustrations, and the scientific technicality of the language displayed in the VA. Non-specialists can appropriate knowledge in a more tangible way, especially with the evolution of social networks, and the ability to share VAs in the digital environment, strengthening the exponential transmission of information and the recall of data afterwards.

The main limitation of the study was the small sample size. In addition, the study did not analyze possible confounding variables, such as the respondent’s age, sex, and occupation, which may influence the aesthetic perception of VAs.

Based on the results of this study, VA editors and scientific journal designers may use the identified aesthetic preferences as a guide when constructing the VA layout. Possible practical implications include: a) optimizing the time spent on constructing the VA layout, with a rational direction of efforts towards the most influential components, considering the complexity of “making it visual”; b) recognizing the target audience facilitates the creation of a personalized design, in line with trends, ensuring the credibility and trust of readers, without significant changes to the journal’s visual identity; c) familiarity with aesthetic preferences allows for standardization of the layout, as well as gradual testing of different VA styles and future research on the subject; d) aesthetic choices guided by readers’ preferences may impact interest in the article, data dissemination, knowledge transmission, and also influence the reading of other articles in the journal.

We suggest that the scientific community develop more comprehensive and analytical studies assessing the impact of using the most relevant visual components in the construction of different VAs concerning the number of views and downloads of the article. Thus, instead of comparing the VA to the written abstract for the abovementioned purpose, it will be possible to compare different VA designs for the same scientific article, enabling VA editors to adapt to readers’ preferences, for a comprehensive dissemination of information and strengthening of the scientific community.

## Conclusion

Visual Abstracts remain little known and are a trend among both national and international journals. The construction of a VA is divided into several components, in terms of visual and written aesthetics, message comprehension, and interest capture, all of which may influence the aesthetic perception of physicians and medical students. Highlights include the detailing of information, layout color, and the use of illustrations, with an aesthetic preference for applying these components through icons, in an organized structure, with textual objectivity and clarity of colors.
