# Supplementary material for: Perceptions related to the layout of Visual Abstracts among physicians and medical students
Source: J Bras Nefrol. 2024 Dec 16;47(2):e20240146. doi: 10.1590/2175-8239-JBN-2024-0146en (PMC11649198; doi:10.1590/2175-8239-JBN-2024-0146en)
Supplement: Translation

# Translation: Percepções relacionadas ao layout de Visual Abstracts por médicos e estudantes de medicina

**DOI:** 10.1590/2175-8239-JBN-2024-0146pt

**Authors:** Pedro Cesana Portugal, José A. Moura-Neto

**Affiliations:** 1 Escola Bahiana de Medicina e Saúde Pública, Salvador, BA, Brasil.

**Keywords:** Resumos, Percepção Visual, Estética, Cor, Ilustração Médica

## Abstract

**Introdução:**

O Visual Abstract corresponde a uma síntese visual das informações mais relevantes, apresentadas como infográfico, de um artigo científico. Apesar da utilização crescente de Visual Abstracts por periódicos no mundo, ainda há escassez de estudos que avaliem os elementos que os compõem para orientar sua elaboração.

**Objetivo:**

O objetivo principal deste estudo é identificar as percepções estéticas dos componentes de Visual Abstracts por médicos e estudantes de medicina.

**Métodos:**

Estudo transversal, por meio da aplicação de questionário virtual enviado por e-mail para uma amostra de conveniência formada por médicos e estudantes de medicina. Dados analisados por estatística descritiva, utilizando-se médias e desvio-padrão ou mediana e intervalo interquartil, a depender do tipo de distribuição da variável. Variáveis categóricas apresentadas em números absolutos e relativos.

**Resultado:**

A amostra da pesquisa foi composta principalmente por estudantes de medicina (65%), do sexo feminino (57,2%), com mediana de idade de 23,5 (IIQ 21-42,25). A maioria dos respondentes declarou desconhecimento prévio sobre Visual Abstracts (61,7%). Das variáveis analisadas, foram preferidos ícones (56,7%), no estilo monocromático (36,7%), em 2^a^ dimensão (81,1%), e layout com detalhamento moderado (56,7%), cor “original” (91,7%) e estruturado IMRD (73,9%).

**Conclusão:**

Diversos componentes visuais influenciam a percepção estética de médicos e estudantes de medicina em relação aos Visual Abstracts, com destaque para a objetividade textual, clareza das cores e a utilização de ícones.

## Introdução

O Visual Abstract (VA), também conhecido como Resumo Visual, corresponde a uma síntese visual das informações mais relevantes, apresentadas como infográfico, de um artigo científico, com o intuito de transmitir uma mensagem que seja rapidamente manuseada e compreendida. O VA não tem o objetivo de substituir a leitura do artigo, e sim ajudar o leitor na tomada de decisão entre prosseguir ou não com sua leitura integral^1,2,3,4^.

Apesar de sua crescente utilização por periódicos internacionais, os estudos ainda são raros e restringem-se à compreensão dos VAs na disseminação do conhecimento. Desde 2016, o Annals of Surgery publica VAs. No Brasil, o Brazilian Journal of Nephrology foi pioneiro no uso da ferramenta, tendo iniciado ainda em 2018. Entretanto, há escassez de informações acerca dos componentes do layout, design visual e da percepção estética dos usuários que possam orientar editores e autores na elaboração de VAs.

O VA pode ser elaborado a partir de diversos elementos, como o sumário dos resultados, título, layout, jornal de publicação e o “making it visual”, o qual corresponde ao uso de ícones, pictogramas e imagens no design, tornando-o mais ilustrativo. Esse aspecto visual, frequentemente considerado como a parte mais complexa e desafiadora na construção do VA, devido à complexidade de escolha entre cores e formatos adequados, ainda carece de um padrão estético definido a ser utilizado entre os editores e autores de VAs. Desse modo, periódicos planejam o “making it visual” sem garantir previamente uma estética visual favorável, podendo resultar em percepções estéticas conflitantes ao estilo do VA, tornando-o potencialmente menos atrativo e menos eficaz em engajar os leitores com o artigo integral^3,4,5,6,7,8,9^.

O objetivo principal deste estudo é identificar as percepções estéticas dos componentes dos layouts de um VA entre médicos e estudantes de medicina, quando expostos a diferentes designs visuais. Os objetivos secundários são identificar o conhecimento prévio da amostra sobre VA e reconhecer os componentes visuais do layout que mais influenciam a escolha do padrão estético de um VA.

## Métodos

### Desenho e Amostra

Este foi um estudo transversal, que incluiu amostragem do tipo conveniência, realizado entre fevereiro a julho de 2022, na Escola Bahiana de Medicina e Saúde Pública (EBMSP). Foram incluídos estudantes de medicina e médicos, com idade mínima de 18 anos, e excluídos aqueles que não preencheram o Termo de Consentimento Livre e Esclarecido (TCLE).

### Coleta de Dados

Dados coletados por meio de formulário com preenchimento único, aplicado virtualmente pela plataforma “Google Forms”, disponibilizado para preenchimento através do e-mail estudantil de estudantes de medicina, do 1° ao 12° semestre, e médicos professores da EBMSP. A coleta foi realizada após aprovação do Conselho de Ética e Pesquisa. Em seguida, foi realizada avaliação detalhada dos dados armazenados em uma planilha do Microsoft Excel. A revisão e alimentação dos bancos de dados foram feitas unicamente pelos pesquisadores responsáveis pelo estudo.

O formulário foi criado pelos próprios pesquisadores. O VA que serviu como modelo base foi criado pelo próprio pesquisador do estudo, utilizando VA publicado pelo Brazilian Journal of Nephrology (BJN) em 2021 (43(2):228-35), com a aprovação dos editores do periódico (Figura 1). A partir dele, a cor utilizada foi denominada “Original”; o ícone, “Monocromático”; dimensionalidade em 2D; e fonte da letra Arial. O layout foi definido como “Não estruturado”, pela ausência da estrutura Introdução – Metodologia – Resultados – Discussão/Conclusão (IMRD) nitidamente descrita; a borda da caixa que continha as informações foi denominada “Retangular”, pela presença de vértices; o número de divisões do conteúdo do VA, “Quádruplo”, pela presença de quatro subdivisões. A utilização de abreviatura de termos médicos, como “Com Abreviaturas”; o layout do rodapé utilizado, “Base no BJN e Clinical Journal of the American Society of Nephrology (CJASN)”, já que esses periódicos tendem a utilizar esse padrão na construção do VA e a divisão do detalhamento foi a partir da Escala Likert (escala de natureza ordinal com descrições verbais contendo diferentes níveis de opiniões).

**Figura 1.** Visual Abstract utilizado para servir como modelo base, publicado no Brazilian Journal of Nephrology em 2021; 43(2):228-35.

A modificação e (re)criação do design do VA foi feita no PowerPoint. A partir dele, foi adquirido o código Hexadecimal das cores e utilizado o aplicativo Adobe Color para modificar a cor do resumo visual. A alteração teve como base a harmonia das cores sugeridas pelo aplicativo, optando-se pelo padrão “Análogo”, ou Monocromático”, ou “Sombra”. O “Análogo” corresponde ao uso de cores adjacentes na roda de cores; o “Monocromático”, em utilizar uma única cor, com variações de saturação e brilho; o “Sombra”, em utilizar cinco cores com a mesma matiz e saturação, mas brilho diferente^10,11^.

Os ícones foram escolhidos através dos websites “Flaticons”, “Noun Project” e “Icon Finder”, e a transformação em 3D através do site “Tinkercad”. O estilo do ícone variou entre “Monocromático”, “Colorido Plano”, “Colorido Contornado”. O “Monocromático” ou “Glyph icon” é todo preto, de coloração única. O “Colorido plano”, ou “Flat icon”, é um estilo que utiliza combinação de cores diferentes. O estilo “Colorido contornado”, ou “Lineal color”, é composto por traços e preenchido por cores^12,13^.

As imagens foram escolhidas através dos websites “Freepik” e “Istock”, variando entre imagem em pixel e em vetor. A imagem em pixel é composta por pixels, que são a unidade de medida padrão da imagem digital, visualizado ao aproximarmos a imagem da visão. A imagem em vetor é reproduzida por pixel, mas não construída por ele, e sim por linhas, curvas e formas preenchidas^14^.

A escolha das opções do rodapé teve como base os periódicos, internacionais e nacionais, pioneiros no uso do VA, como o BJN, que opta por colocar a conclusão do artigo de forma mais descritiva, o Annals of Surgery, que opta por colocar a referência do artigo no rodapé, e o New England Journal of Medicine (NEJM), que sumariza a conclusão do artigo, de forma sucinta, no rodapé do VA.

### Análise Estatística

Todas as variáveis foram analisadas quanto ao seu tipo de distribuição, se gaussiana ou não. Tal análise envolveu observações do grau de similaridade das curvas de distribuição de frequências com a curva de Gauss, mas também a utilização dos testes de Shapiro-Wilk e/ou Kolmogorov-Smirnov.

A análise dos dados ocorreu através da estatística descritiva, utilizando-se médias e desvio-padrão ou mediana e intervalo interquartil, a depender do tipo de distribuição da variável, e as variáveis categóricas foram apresentadas em números absolutos e relativos.

### Aspectos Éticos

Este estudo foi aprovado pelo Comitê de Ética em Pesquisa da EBMSP no dia 11 de fevereiro de 2022, parecer 5.238.369. Foi utilizado o TCLE para coleta dos dados.

## Resultados

Foram obtidas 181 respostas ao questionário entre fevereiro e julho de 2022. Entre elas, restaram 180 (99,4%) respostas para análise, após aplicação dos critérios de exclusão. A maioria da amostra (57,2%) era do sexo feminino, a mediana das idades dos médicos e estudantes de medicina foi de 23,5 (IIQ 21–42,25). A variável ocupação, avaliada através da dicotomia médico e estudantes de medicina, teve a maioria (65%) representada por estudantes (Tabela 1).

**Tabela 1.** Características sociodemográficas

Variável	Amostra (N = 180)
**Sexo**	
Masculino	77 (42,8%)
Feminino	103 (57,2%)
**Idade (em anos)**	23,5 (21-42,25)
**Ocupação**	
Médico	63 (35%)
Estudante de Medicina	117 (65%)

Fonte: As variáveis qualitativas foram expressas em absoluto (porcentagem válida) e a quantitativa expressa em mediana (intervalo interquartil-IIQ).

A maioria (61,7%) dos respondentes desconhecia VAs. Dentre os grupos, a maioria dos médicos tinha conhecimento da ferramenta (59,4%), enquanto cerca de um quarto dos estudantes (26,5%) conhecia previamente VA (Tabela 2). As percepções estéticas foram identificadas por meio de diversas variáveis, sendo as preferidas aquelas que contemplavam o título apresentado na forma de tema do artigo (39,4%), o layout no padrão original (91,7%), com preferência do uso de ícones em relação ao uso de imagens (56,7%), e preferência para o ícone no estilo monocromático (36,7%) e bidimensional (81,1%).

**Tabela 2.** Conhecimento prévio sobre um Visual Abstract

Variável	Amostra (N = 180)
**Estudante de Medicina**	117 (65%)
Conhece VA	31 (26,5%)
Desconhece VA	86 (73,5%)
**Médico**	63 (35%)
Conhece VA	38 (60,3%)
Desconhece VA	25 (39,7%)

Fonte: As variáveis qualitativas foram expressas em número absoluto (porcentagem válida).

Além disso, teve maior relevância o layout estruturado IMRD (73,9%), sem abreviaturas (55,6%), com fonte de letra Arial (46,1%) e detalhamento moderado (56,7%). Ademais, tiveram maior expressão a quantidade de três ou mais divisões (47,2%), com a borda da caixa de formato retangular (58,9%) e o rodapé com base no NEJM (43,9%) (Tabela 3). O componente considerado mais influente no padrão estético para o design visual do VA foi o detalhamento de informações (83,3%), seguido pela cor do layout (81,1%), pelo uso de ícones e imagens (71,7%), pelo design de apresentação do título (70%) e pela estrutura do layout (69,4%) (Tabela 4).

**Tabela 3.** Percepções estéticas dos componentes do layout de um Visual Abstract

Variável	Amostra (N = 180
**Design de apresentação do título**	
Pergunta de investigação	59 (32,8%)
Título do artigo	44 (24,4%)
Tema do artigo	71 (39,4%)
Sem preferência	06 (3,3%)
**Cor do layout**	
Original	165 (91,7%)
Sombra	05 (2,8%)
Análogo	06 (3,3%)
Monocromático	03 (1,7%)
Sem preferência	01 (0,6%)
**Estilo do ícone**	
Monocromático	66 (36,7%)
Colorido plano	56 (31,1%)
Colorido contornado	51 (28,3%)
Sem preferência	06 (3,3%)
**Outros**	
Monocromático ou Colorido contornado	01 (0,6%)
**Dimensão do ícone**	
Versão 2D	146 (81,1%)
Versão 3D	23 (12,8%)
Sem preferência	11 (6,1%)
**Uso de ícones e outras imagens**	
Ícone	102 (56,7%)
Imagem em pixel	15 (8,3%)
Imagem em vetor	59 (32,8%)
Sem preferência	04 (2,2%)
**Fonte da letra**	
Arial	83 (46,1%)
Consolas	03 (1,7%)
Courier New	06 (3,3%)
Georgia	12 (6,7%)
Times New Roman	25 (13,9%)
Verdana	28 (15,6%)
Sem preferência	23 (12,8%)
**Estrutura IMRD**	
Não estruturado	25 (13,9%)
Estruturado	133 (73,9%)
Sem preferência	22 (12,2%)
**Borda da caixa fechada**	
Retangular	106 (58,9%)
Arredondada	58 (32,2%)
Sem preferência	16 (8,9%)
**Quantidade de divisões**	
Única	16 (8,9%)
Dupla	69 (38,3%)
Três ou mais	85 (47,2%)
Sem preferência	09 (5%)
**Outros**	
Divisão tripla	01 (0,6%)
**Utilização de abreviaturas**	
Com abreviaturas	68 (37,8%)
Sem abreviaturas	100 (55,6%)
Sem preferência	10 (5,6%)
**Outros**	
Com abreviação, desde que exista uma legenda embaixo explicando	02 (1,1%)
**Detalhamento de informações**	
Grau 1 = Apenas informações essenciais	09 (5%)
Grau 2π	19 (10,6%)
Grau 3 = Moderado detalhamento	102 (56,7%)
Grau 4ππ	34 (18,9%)
Grau 5 = Máximo detalhamento possível	11 (6,1%)
Sem preferência	05 (2,8%)
**Design do rodapé**	
Base no BJN* e CJASN**	65 (36,1%)
Base no Annals of Surgery***	32 (17,8%)
Base no NEJM	79 (43,9%)
Sem preferência	03 (1,7%)
**Outros**	
Baseado no BJN e CJASN, com conclusão resumida	01 (0,6%)

Fonte: Questionário elaborado pelos autores. As variáveis qualitativas foram expressas em número absoluto (porcentagem válida). πEstágio intermediário entre o Grau 1 e Grau 3 ππ Estágio intermediário entre Grau 3 e Grau 5 *Brazilian Journal of Nephrology. **Clinical Journal of the American Society of Nephrology. ***New England Journal of Medicine.

**Tabela 4.** Componentes visuais que mais influenciam a escolha do padrão estético do design de um Visual Abstract

Variável	Amostra (N = 180)
Design de apresentação do título	126 (70%)
Cor do layout	146 (81,1%)
Estilo do ícone	84 (46,7%)
Dimensão do ícone	76 (42,2%)
Uso de ícones e outras imagens	129 (71,7%)
Fonte da letra	113 (62,8%)
Estrutura IMRD	125 (69,4%)
Borda da caixa fechada	56 (31,1%)
Quantidade de divisões	101 (56,1%)
Detalhamento de informações	150 (83,3%)
Design do rodapé	87 (48,3%)
Nenhuma	01 (0,6%)
**Outro**	
Conteúdo	01 (0,6%)

Fonte: Questionário elaborado pelos autores. As variáveis qualitativas foram expressas em número absoluto (porcentagem válida).

## Discussão

O presente estudo demonstrou diversos componentes considerados influentes para o padrão estético do VA. Apesar do desconhecimento acerca do “making it visual”, o ramo científico tem focado principalmente na comparação do VA com o resumo escrito para disseminar informações. Um estudo de 2017 avaliou que o uso do VA aumentou o número de impressões (vezes em que o tweet foi visualizado), de retweets (republicação do tweet aos próprios seguidores) e da média de inspeções ao artigo original em comparação ao uso apenas do título do artigo^7^. Outro estudo, publicado em 2020, demonstrou aumento de impressões, retweets e cliques com o uso do VA em relação ao uso Text Abstract (captura de tela do abstract presente no PubMed)^5^.

O VA vem sendo cada vez mais utilizado por periódicos com alto Fator de Impacto, como NEJM, Annals of Surgery, CJASN, Journal of the American Society of Nephrology (JASN) e Kidney360^4^. Entretanto, não há um padrão estabelecido da qualidade do design visual do VA, gerando uma variedade de construções estéticas sem respaldo técnico consistente. Nesse sentido, o ineditismo deste estudo está na abordagem da percepção estética dos VAs e reconhecimento dos componentes visuais, contribuindo para direcionar editores e autores na elaboração de um design gráfico de layout mais atrativo para leitores.

Os layouts de um VA combinam diferentes elementos para garantir sua capacidade transmissora e de memorização de conteúdo. Com base nas variáveis do presente estudo, o detalhamento de informações foi considerado o componente de maior influência para o padrão estético, com preferência pelo nível moderado de detalhamento. O equilíbrio na quantidade de informação no VA é importante para garantir clareza e organização do layout, e pode impactar a percepção do leitor e a compreensão dos resultados do estudo^15,16^. A construção de um layout com narrativa visual associada à hierarquização de informações em uma sequência lógica, intuitiva e fluida – por exemplo, da esquerda para a direita e de cima para baixo – contribui para o desenvolvimento da memória visual e textual. Esses são dois dos quatro estilos de aprendizagem do modelo VARK – acrônimo dos termos em inglês que se referem às modalidades de aprendizagem Visual, Auditiva, Leitura/Escrita e Cinestésica.

A cor do layout foi o segundo componente de maior influência para o design visual do VA, com preferência pela cor “Original”, que corresponde à coloração padrão e paleta de cores do modelo base de VA utilizado pelo periódico. A cor influencia o reconhecimento de informação, a legibilidade e torna atrativo visualmente. Cores podem ser utilizadas em contraste para evidenciar uma informação e em associação (a exemplo do vermelho, para enfatizar um alerta) com o objetivo de atrair a atenção do público a um dado de maior importância, fortalecendo a memória de longo prazo.

A preferência pela coloração “Original” pode ser consequência da exposição constante ao layout do modelo base durante o questionário ou se dar pela simplicidade da combinação das colorações utilizadas ou reduzida habilidade teórico-prática em design pelos autores, dificultando a construção do VA pautada na harmonia das cores^17^.

Houve preferência pelo uso de ícones em relação ao uso de imagens, sendo considerado o terceiro componente estético mais relevante. A utilização de ícones e imagens, de modo geral, torna o design mais ilustrativo, captando a atenção do indivíduo e aprimorando a capacidade de recordação. No geral, os ícones são menores e mais diversos, com diferentes formas e estilos, o que pode implicar maior possibilidade de escolhas que se adequem à construção de um design estético do VA; entretanto, na maioria dos VAs há ícones em seu layout tradicional, o que representa um viés aos respondentes já acostumados com o padrão^4,18,19^.

Dentre os ícones, a principal escolha foi pelo estilo “Monocromático” (36,7%). A diferença entre o “Colorido plano” e o “Colorido contornado”, entretanto, consiste apenas na presença de um contorno que realce o perímetro do pictograma. Entretanto, quando somadas, a preferência pela utilização de ícones coloridos, plano ou contornado, corresponde a 59,4%, o que pode sugerir preferência pelo uso de cores em VAs. Em relação às imagens, a versão em vetor teve maior preferência, possivelmente devido à capacidade de alcançar formas mais próximas da realidade e manter a qualidade da imagem, independentemente do tamanho^14^.

A estrutura do layout foi considerada o quinto componente mais influente para o padrão estético de um VA. Layouts com simetria e proporcionalidade geram sensação de ordem e equilíbrio, o que reduz a sobrecarga cognitiva e simplifica a fixação da mensagem, com consequente facilidade de relembrar/evocar a informação. A descrição nítida do IMRD, assim como o layout com três ou mais divisões, distribui e organiza melhor as ideias, direcionando o leitor a pontos-chave no texto, impactando na eficácia de transmitir informações, principalmente, considerando que a maioria da amostra é formada por estudantes ainda em processo de desenvolvimento da leitura e do interesse científico,,^15,20,21^. Apesar disso, juntamente com a fonte da letra, apresentaram a maior porcentagem de “Sem Preferência”, o que torna discutível a relevância estética desses subtipos.

O título preferível para a apresentação do VA foi em formato de “Tema do Artigo”. Um título visualmente mais simples pode facilitar a compreensão da mensagem, deixar o layout mais acessível e direto, indo em paralelo com a influência do detalhamento de informações na estética do design visual^15,16^.

Diferentes layouts comunicam-se com públicos-alvo diferentes por meio da disposição dos componentes, escolha das ilustrações e tecnicidade científica da linguagem exposta no VA. Não especialistas podem apropriar-se do conhecimento de forma mais tangível, ainda mais com a evolução das redes sociais e capacidade de compartilhamento de VAs no meio digital, fortalecendo uma transmissão exponencial das informações e evocação do dado *a posteriori*.

A principal limitação do estudo foi o pequeno tamanho amostral. Além disso, o estudo não analisou possíveis variáveis de confusão, como idade, sexo e ocupação do respondente, que podem influenciar na percepção estética dos VAs.

A partir dos resultados deste estudo, editores de VAs e designers de periódicos científicos podem utilizar-se das preferências estéticas identificadas para se orientar durante a construção do layout do VA. Podem ser consideradas possíveis implicações práticas: a) a otimização do tempo de construção do layout do VA, com direcionamento racional dos esforços para os componentes mais influentes, considerando a complexidade do “making it visual”; b) o recon­hecimento do público-alvo facilita a elaboração de um design personalizado, alinhado às tendências, garantindo a credibilidade e confiança dos leitores, sem alterações significativas na identidade visual do periódico; c) a familiaridade com as preferências estéticas permitem a padronização do layout, assim como a testagem gradativa de diferentes estilos de VAs e pesquisas futuras sobre o tema; d) as escolhas estéticas guiadas pelas preferências dos leitores podem impactar o interesse no artigo, a disseminação dos dados, a transmissão do conhecimento e, ainda, influenciar a leitura de outros artigos do periódico.

Sugerimos à comunidade científica a elaboração de trabalhos mais abrangentes e analíticos que avaliem o impacto da utilização dos componentes visuais considerados mais relevantes na construção de diferentes VAs no que se refere ao número de visualizações e downloads do artigo. Desse modo, ao invés de comparar o VA ao resumo escrito para o objetivo acima, será possível comparar diferentes designs de VAs para um mesmo artigo científico, possibilitando que editores de VA se adaptem às preferências dos leitores, para uma abrangente disseminação de informação e fortalecimento da comunidade científica.

## Conclusão

Visual Abstracts são ainda pouco conhecidos e uma tendência entre periódicos nacionais e internacionais. A construção de um VA divide-se em diversos com­ponentes, no âmbito da estética visual e escrita, da compreensão da mensagem e da captação de interesse, que podem influenciar a percepção estética de médicos e estudantes de medicina. Destacam-se o detalhamento de informações, a cor do layout e a utilização de ilustrações, com preferência estética para aplicação desses componentes através de ícones, em uma estrutura organizada, com objetividade textual e clareza de cores.
